# Diagnostic and psychopharmacotherapy in the general practictioner practice

**DOI:** 10.1192/j.eurpsy.2023.1758

**Published:** 2023-07-19

**Authors:** F. H. Kraxner

**Affiliations:** Center of psychiatry and psychotherapy, Hospital of Affoltern am Albis, Affoltern am Albis, Switzerland

## Abstract

**Introduction:**

Due to the often long-standing and extensive doctor-patient relationship, family doctors have special access to the mental state of their patients. They are often the first point of contact, and consequently the treatment of depression often begins in the GP’s practice or even takes place entirely there. This requires dedicated knowledge on the part of the general practitioner, especially with regard to diagnostic criteria and treatment.

**Objectives:**

The aim of this article is to describe the basic diagnostic process for the general practitioner’s practice, to give advice on the indication and implementation of psychopharmacological interventions, and to present the results. This overview summarises the most relevant connections to the diagnosis, assessment of the severity and psychopharmacotherapy of depression in general practice.

**Methods:**

The following therapy algorithms and remarks are essentially based on the treatment recommendations of the Swiss Society for Psychiatry and Psychotherapy (SGPP) and the Swiss Society for Anxiety and Depression (SGAD) as well as the German S3 guideline of the German Society for Psychiatry and Psychotherapy, Psychosomatics and Neurology (DGPPN).

**Results:**

Family doctors play a central role in the treatment of depressive disorders. They are often the first point of contact for patients with depression and in about 40 percent of cases even the only contact point. The likelihood of developing a depressive episode in the course of a lifetime is 10 to 15 percent globally. Evaluations by the World Health Organisation WHO show that 9 to 23 percent of people with chronic illnesses have depression as a concomitant illness. A cross-sectional epidemiological study in Germany showed that 60 percent of patients in general medical care were not treated with antidepressants and/or psychotherapy in accordance with guidelines. In Switzerland, about half of the antidepressants are currently prescribed by general practitioners. Image 1 shows a detailled overview (in German) of the current medication.

**Image:**

**Figure:**
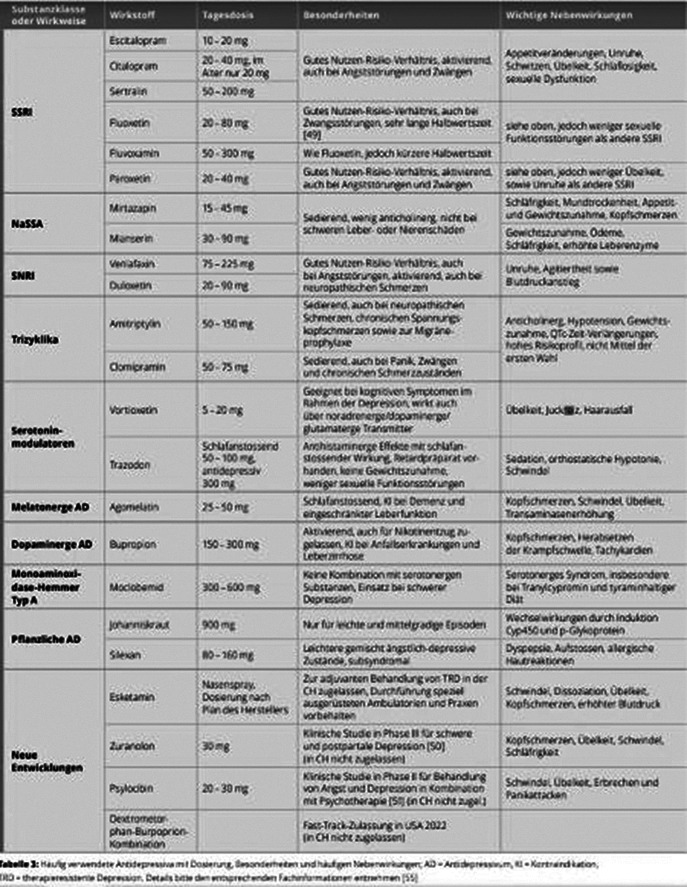

**Conclusions:**

Specialists in general internal medicine have a central role in recognition and treatment of depressive syndromes. Somatic causes can be ruled out by means of physical examination, laboratory and ECG/EEG/imaging. Mild and moderate depressive episodes can be treated by psychoeducation, counselling and medication. If the symptoms are mild, psychosocial support or psychotherapy alone can be considered. If acute suicidal tendencies or psychotic symptoms are identified, emergency symptoms, emergency admission to a psychiatric hospital should be considered.

The presence of other psychiatric comorbidities, resistance to therapy or complex psychiatric medication necessitate referral to outpatient specialists. Metabolic and cardiovascular side effects and interactions between psychopharmacological and internal medicine must be considered.

**Disclosure of Interest:**

None Declared

